# *Fretibacterium* sp. human oral taxon 360 is a novel biomarker for periodontitis screening in the Japanese population

**DOI:** 10.1371/journal.pone.0218266

**Published:** 2019-06-19

**Authors:** Thatawee Khemwong, Hiroaki Kobayashi, Yuichi Ikeda, Takanori Matsuura, Takeaki Sudo, Chihiro Kano, Ryo Mikami, Yuichi Izumi

**Affiliations:** 1 Department of Oral Diagnosis, Naresuan University, Phitsanulok, Thailand; 2 Department of Periodontology, Graduate School of Medical and Dental Sciences, Tokyo Medical and Dental University, Tokyo, Japan; 3 Oral Care Perio Center, Southern TOHOKU General Hospital, Fukushima, Japan; University of the Pacific, UNITED STATES

## Abstract

**Background:**

Periodontitis is a common inflammatory disease, leading to bone destruction and tooth loss. Screening for periodontitis is important in preventing the progress of this disease. Various types of bacteria have been examined as potential screening targets, but only culturable pathogenic bacteria have been considered candidates. Recently, the various uncultivable bacteria have been identified in microbiome studies, but the value of these bacteria in periodontitis screening remains unknown.

**Objectives:**

The aim of this study was to evaluate the diagnostic use of uncultivable bacteria *Fretibacterium* sp. HOT 360 and *TM7* sp. HOT 356 for periodontitis screening in the Japanese population.

**Material and methods:**

Stimulated saliva samples were collected from 217 participants (periodontitis group, *n* = 157; healthy group, *n* = 60). The two uncultivable bacterial species selected were: *Fretibacterium* sp. human oral taxon 360 (*Fretibacterium* sp. HOT 360) and *TM7* sp. human oral taxon 356 (*TM7* sp. HOT 356). The levels of these two bacterial species were compared with those of *Porphyromonas gingivalis* (*P*. *gingivalis*), a keystone pathogen in periodontitis. These three species of bacteria were then quantified using qualitative real-time polymerase chain reaction (qPCR) with specific primers and *Taqman* probes. Statistical analysis was performed by SPSS 20.0 software. *P* value was statistically significant at .05.

**Results:**

The populations of uncultivable bacterial species *TM7* sp. HOT 356 and *Fretibacterium* sp. HOT 360 were significantly higher in periodontitis group than in healthy group. Only *Fretibacterium* sp. HOT 360 showed a significantly positive correlation with such periodontal parameters as probing pocket depth (PPD) and bleeding on probing (BOP).

**Conclusion:**

These findings indicate that uncultivable bacteria *Fretibacterium* sp. HOT 360 can be used as a saliva-based diagnostic bacterial biomarker for periodontitis screening.

## Introduction

Periodontitis is a common disease that occurs worldwide. It is an inflammatory condition that affects the periodontium, leading to bone destruction and tooth loss [1]. The disease progresses slowly with no symptoms. Early screening is important for preventing the progression of this disease and improving oral health. Diagnosis of periodontitis is limited to clinical parameters such as loss of attachment and radiography. However, these parameters can be determined only by examination conducted by a dentist. It is necessary to establish an efficient screening method showing the progress of periodontitis [2]. Screening using saliva has recently become a common screening method. Saliva is a diagnostic tool that is established in the medical and dental fields. Collection of saliva is straightforward and non-invasive, and can be performed by an individual in a home setting [2]. Saliva contains various factors including dental biofilm, gingival crevicular fluid [3], and microorganisms [4]. Periodontal pathogenic bacteria in saliva are associated with symptoms of periodontal disease [5].

In periodontitis, periodontopathic bacteria are the most common cause of the host immune response [6]. Several bacteria have been isolated and cultured from the oral cavities of patients with periodontitis. Among these bacteria, those detected with high frequency are called periodontopathogenic bacteria. The major isolated periodontopathogens are *P*. *gingivalis*, *Treponema denticola*, and *Tannerella forsythia*, which colonize subgingival deep pockets [7]. These culturable periodontopathogenic bacteria have been examined for their characteristics, virulence factors, and host-pathogen interactions in infective etiology [8]. Recent advancements in molecular biology have allowed for detailed analysis of uncultivable oral microbiota [9]. More than half of the bacteria in the oral cavity are uncultivable, and their role is unknown [10]. Numerous cultivable periodontopathogens are used as markers of chronic periodontitis [11], but the usefulness of uncultivable periodontopathogens remains unknown. Using our microbiome data [12], existing microbiome reports [13], and systematic reviews [14], we selected two uncultivable candidate bacterial species: *Fretibacterium* sp. HOT 360 and *TM7* sp. HOT 356.

*Fretibacterium* sp. HOT 360 belongs to phylum Synergistetes. Although there are no reports detailing the characteristics of this bacterial species, it is found in high proportion in subgingival dental plaque of patients with chronic periodontitis [13–17]. Candidate division TM7 (TM7) is almost always detected in the human oral cavity [9]. TM7 forms colonies in periodontal subgingival plaque and is associated with periodontal disease [18, 19]. *TM7* sp. HOT 356 (TM7 Clone I025) is detected with high frequency in the subgingival dental plaque of patients with chronic periodontitis [13, 14, 19, 20]. For these reasons, not only red complex bacterial species as *P*. *gingivalis*, *Treponema denticola*, and *Tannerella forsythia*, but also uncultivable bacterial species might be representative as periodontopathogens marker. To measure these uncultivable bacteria, we used 16s rDNA amplification with specific primers and probe by qPCR. Using artificial synthetic gene expression as a standard curve for qPCR, it is possible to accurately quantify the expression of uncultivable bacteria. The aim of this study was to evaluate *Fretibacterium* sp. HOT 360 and *TM7* sp. HOT 356 for use as diagnostic biomarkers in saliva for periodontitis screening.

## Materials and methods

### Recruitment of participants

Overall, 217 patients were recruited between January 2014 and August 2015 from the Department of Periodontology at Tokyo Medical and Dental University (Tokyo, Japan). Written informed consent was obtained from all the enrolled participants. Ethical approval was obtained from the Dental Research Ethics Committee of Tokyo Medical and Dental University (Reference number: 1023).

### Saliva collection and periodontal condition assessment

In this study, we included patients who were more than 20 years old and had been examined by their periodontists. We excluded patients who were administered medications implicated in progression of periodontitis, such as systemic antibiotics, anti-inflammatory drugs, or immunosuppressive drugs, within 3 months of the start of this investigation. Patients who had been diagnosed with diabetes mellites, bleeding disorders and viral infections, such as those with human immunodeficiency virus, human papillomavirus, or hepatitis virus, were also excluded. Intra examiner calibration was performed using a dental model for pocket probing (Nissin 500H-PRO, Nissin, Japan). The examiners evaluated 36 sites in 6 teeth on this dental model. Calibration was accepted if measurement error was within a millimeter at ≥ 90%[21]. Then, all patients were diagnosed by one periodontist (H.K.) and received full-mouth periodontal examination, bleeding on probing (BOP), and probing pocket depth (PPD) assessment at six sites of each tooth. We then obtained periapical digital radiographs (CS7600, Carestream, US). Participants were instructed to abstain from eating, smoking, and performing oral hygiene procedures for at least 2 h before saliva collection. Stimulated whole saliva was collected from each participant after that participant chewed chewing gum (GC, Japan) for 90 s. Participants were classified based on guidelines which were modified from Offenbacher’s classification to identify patients with deep periodontal pockets and bleeding on probing sites [22]. Participants who did not present with ≥4 mm of PPD, and presented with less than 10% of BOP, were categorized into the healthy group (*n* = 60). Participants who presented with ≥4 mm of PPD were categorized into periodontitis group (*n* = 157).

### Confounding factors and assessment of patient symptoms

Symptoms of periodontitis were defined as frequency of pain, gingival bleeding, tooth mobility, dry mouth, malodor, and bruxism. Confounding factors such as oral hygiene behavior, frequency of tooth brushing, duration of tooth brushing, utilization of special appliances, frequency of brush changing, utilization of fluoride supplements, alcohol consumption, and smoking behavior were obtained via structured interviews.

### Extraction of bacterial DNA and qPCR

To separate the debris, samples of stimulated whole saliva were centrifuged at 3000 *g* for 10 min in room temperature. The obtained supernatants were stored at −20°C [23] until extraction of genomic DNA. Genomic DNA was extracted using QIAamp DNA Mini kit (QIAGEN., CA, USA). The sequences for the specific primers and probes used to examine the expression of *P*. *gingivalis* [24], *TM7* sp. HOT 356 [19], and *Fretibacterium* sp. HOT 360 are listed in Table 1. qPCR was performed using Premix Ex Taq (Takara-bio Inc., Shiga, Japan). The protocols were modified. The briefly procedures were demonstrated by one reaction was mixed by firstly 10 μl of Premix Ex Taq (2X). Then, 0.4 μl of 10 μM PCR Forward and Reverse primers were added. After that, 1 μl of 10 μM Taqman probe and 7.2 μl of Nuclease-Free Water (QIAGEN., CA, USA) were added. Finally, 1 μl each sample was added into 96 well qPCR plate before beginning the process. The total volume of one reaction was 20 μl and the final concentration of Forward, Reverse primer and Taqman probe were 0.2, 0.2 and 0.5 μM, respectively. Amplification was performed with Thermal Cycler Dice Real Time System II (Takara-Bio Inc.) by using following parameters; 40 cycles, 95 C° for 10 sec, 95 C° for 5 sec and 60 C° for 30 sec. The standard curve was obtained by 10-fold serial dilutions of artificial synthetic gene which contained the amplified region of sequences of *P*. *gingivalis*, *TM7* sp. human oral taxon 356 and *Fretibacterium* sp. human oral taxon 360. (Eurofin genomics, Tokyo, Japan).

**Table 1:** Specific primer and probe sequences.

Bacterial species	Sequences of primers and probes		Reference
*Porphyromonas gingivalis*	Primer F	5’-TAGCTTGCTAAGGTCGATGG-3’	[24]
	Primer R	5’-CAAGTGTATGCGGTTTTAGT-3’	
	Probe	FAM-TGCGTAACGCGTATGCAACTTGCC-TAMRA	
*TM7* sp. human oral taxon 356	Primer F	5’-TGACTGGGCGTAAAGAGTTG-3’	[19]
	Primer R	5’-TTCGAACAACAAGCTATCGG-3’	
	Probe	FAM-TCGCTCGCTAACTTGACCGCC-TAMRA	
*Fretibacterium* sp. human oral taxon 360	Primer F	5’-GGAAACATTGACGACGCTG-3’	Novel*
	Primer R	5’-CTTAACCCAACATCTCACGAC-3’	
	Probe	FAM-CACCTGTGTATGCTCACTGCCCGAAA-TAMRA	

*16s rDNA gene sequences of these bacterial species were obtained from GenBank (http://www.ncbi.nlm.nih.gov), Human Oral Microbiome Database (http://www.homd.org/), and using the basic local alignment search tool (BLAST).

### Statistical analysis

Statistical analysis was performed using SPSS 20.0 software (SPSS Inc., Chicago, IL). The demographic data as qualitative data were analyzed by Chi-square test. Quantitative data were analyzed by Kruskal–Wallis and Mann–Whitney *U* tests. To demonstrate the proper bacterial biomarker in this study, First, bacterial load data were normalized using Kolmogorov–Smirnov test. After that, differences between groups (healthy vs periodontitis) were analyzed by Kruskal–Wallis and Mann–Whitney *U* tests. Second, to find the correlation between amount of bacterial species and periodontal parameters, as percentage of 4 mm of PPD and BOP, bootstrapped multiple regression were performed. Finally, to demonstrated differences of bacterial loads within each parameter, the bacterial load data were classified into subgroups. These data were analyzed by Kruskal–Wallis and Mann–Whitney *U* tests, in case of non- parametric data. In addition, parametric data were analyzed by one-way analysis of variance (ANOVA). We performed one-way ANOVA with bootstrapped function to examine differences within parameters in these groups. *P* value was considered statistically significant at 0.05.

## Results

### Characteristics of participants

Demographic and confounding factors, as gender systemic disease, age, oral hygiene behavior, patient symptoms, alcohol consumptions, smoking behavior and remaining teeth, were compared between healthy and periodontitis groups. Moreover, the periodontal parameters as percentage of PPD and BOP were analyzed. These results are shown in Table 2. We also compared demographic factors, such as gender and presence of systemic diseases, and found significant association between genders and the presence of periodontitis (*x*^2^ = 4.77, *P* = 0.04). Similarly, a significant difference in quantitative data as age was observed between the periodontitis and healthy groups. In contrast, the presence of systemic diseases was not significantly associated with development of periodontitis. Moreover, we did not observe significant differences in the number of remaining teeth between patients with periodontitis. Confounding factors, such as oral hygiene behavior, smoking history, and alcohol consumption were not significantly associated with the development of periodontitis. However, bleeding during brushing and development of tooth mobility were significantly associated with the presence of periodontitis (*x*^2^ = 11.12 and *x*^2^ = 13.73, *P* = 0.02 and 0.01 respectively). The results of PPD and BOP assessment are shown in Table 2. We found significant differences in percentages of ≥4 mm and ≥ 6 mm PPD, and in BOP, between healthy participants and those with periodontitis.

**Table 2:** Demographic data showing confounding factors and clinical periodontal parameters in healthy participants and those with chronic periodontitis.

	Healthy	Periodontitis	*P*-value
	(n = 60)	(n = 157)	
**Gender**			
**Male**	17 (7.8%)	70 (32.3%)	0.04*
**Female**	43 (19.8%)	87 (40.1%)	
**Systemic diseases**			
**Healthy**	39 (18.0%)	120 (55.3%)	0.18
**Hypertension**	19 (8.7%)	29 (13.4%)	
**Heart diseases**	1 (0.5%)	6 (2.7%)	
**Heart diseases and hypertension**	1 (0.5%)	2 (0.9%)	
**Age**	65 (56.75,72.25)	61 (49.0,69.0)	0.05*
**Oral hygiene behavior**			
**- How often do you brush your teeth per day (times)?**	2 (2, 3)	2 (2, 3)	0.67
**- How long do you brush your teeth in one time (minutes)?**	5 (3, 10)	5 (3, 10)	0.89
**- Do you use special appliances (such as dental floss or interproximal brush)?**			
**Always**	43 (19.8%)	101 (46.5%)	0.50
**Sometimes**	9 (4.2%)	34 (15.7%)	
**Never**	8 (3.7%)	22 (10.1%)	
**- How often do you change your toothbrush?**			
**Every 2 weeks**	6 (2.8%)	12 (5.5%)	0.67
**Every 1 month**	33 (15.2%)	70 (32.3%)	
**Every 2 months**	12 (5.5%)	45 (20.7%)	
**More than 3 months**	9 (4.1%)	30 (13.9%)	
**- Do you use fluoride supplements (such as toothpaste and mouth wash)?**			
**Always**	37 (17.1%)	72 (33.2%)	0.50
**Sometimes**	5 (2.2%)	19 (8.8%)	
**Never**	18 (8.3%)	66 (30.4%)	
**Patient symptoms**			
**- Do you feel pain around teeth or gums?**			
**Always**	5 (2.3%)	18 (8.3%)	0.58
**Sometimes**	15 (6.9%)	46 (21.2%)	
**Never**	40 (18.4%)	93 (42.9%)	
**- Do you bleed during brushing?**			
**Always**	1 (0.5%)	18 (8.3%)	0.02*
**Sometimes**	8 (3.6%)	41(18.9%)	
**Never**	51 (23.5%)	98 (45.2%)	
**- Do you feel tooth mobility?**			
**Always**	2 (0.9%)	33 (15.2%)	0.01*
**Sometimes**	5 (2.3%)	24 (11.1%)	
**Never**	53 (24.4%)	100 (46.1%)	
**- Do you feel dry in your month?**			
**Always**	6 (2.8%)	22 (10.1%)	0.72
**Sometimes**	16 (7.4%)	41 (18.9%)	
**Never**	38 (17.5%)	94 (43.3%)	
**- Do you have oral malodor?**			
**Always**	5 (2.3%)	16 (7.4%)	0.85
**Sometimes**	22 (10.1%)	52 (24.0%)	
**Never**	33 (15.2%)	89 (41.0%)	
**- Do you have bruxism?**			
**Always**	10 (4.6%)	25 (11.5%)	0.51
**Sometimes**	4 (1.8%)	19 (8.8%)	
**Never**	46 (21.2%)	113 (52.1%)	
**Alcohol consumption per a week (times)**	0 (0, 2)	1 (0, 4)	0.24
**Smoking behavior**			
**Currently smoking**	2 (0.9%)	17 (7.8%)	0.08
**Stopped smoking**	11 (5.0%)	41 (18.9%)	
**Never smoked**	47(21.7%)	99 (45.7%)	
**Remaining teeth (teeth)**	26 (22, 27.8)	26 (22, 28)	0.57
**Periodontal clinical parameters**			
**Probing pocket depth (% of PPD)**			
**≥ 4 mm of PPD**	0 (0,0)	4.76 (1.54, 10.7)	<0.01*
**≥ 6 mm of PPD**	0 (0,0)	0.98 (0, 3.85)	<0.01*
**Bleeding on probing (% of BOP)**	0 (0,0.61)	6.66 (1.12,16.7)	<0.01*

Qualitative data were analyzed using Chi-square test and log linear analysis. Results are shown as percentage.

Quantitative data and non-parametric data were analyzed by Kolmogorov-Smirnov and Kruskal-Wallis tests, respectively. Results are shown as median, 1^st^ quartile and 3^rd^ quartile

* indicates statistical significance.

**Fig 1:**
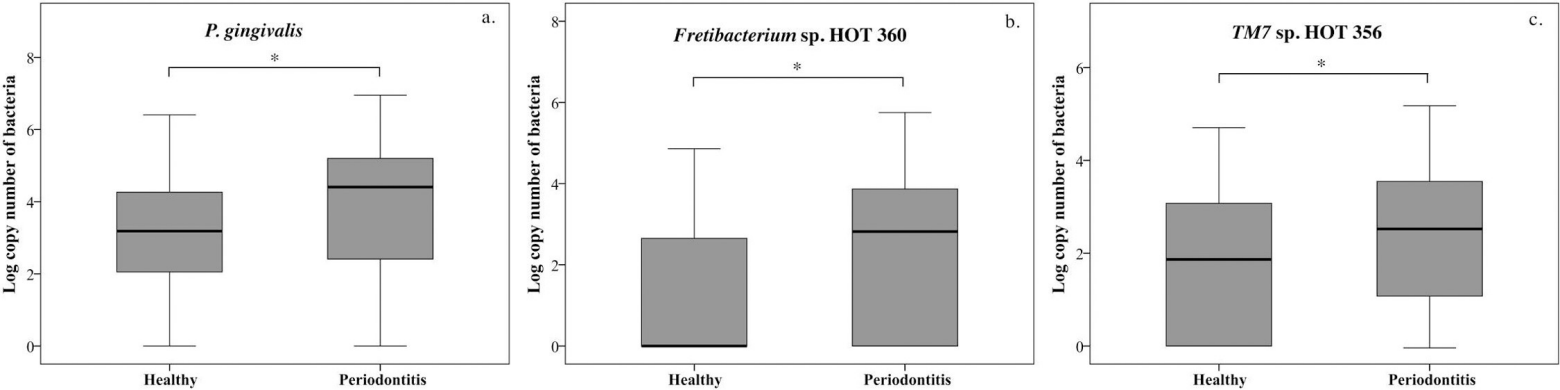
Bacterial load in healthy participants and those with periodontitis. Box plot graphs show the levels of periodontopathogens evaluated in this study. The copy numbers of *P*. *gingivalis* (a.), *Fretibacterium* sp. HOT 360 (b.) and *TM7* sp. HOT 356 (c.) were significantly higher in participants with chronic periodontitis than in healthy participants. *P < 0.05.

We next compared bacterial loads of *P*. *gingivalis*, *Fretibacterium* sp. HOT 360, and *TM7* sp. HOT 356 in healthy participants and those with periodontitis; the results are shown in Fig 1. The box plot graphs in Fig 1 showed that the copy numbers of *P*. *gingivalis* (a), *Fretibacterium* sp. HOT 360 (b), and *TM7* sp. HOT 356 were significantly higher in patients with periodontitis than in healthy participants (*P* < 0.05).

### Correlations between bacterial loads and periodontal parameters

We performed a multiple regression analysis to examine correlations between the bacterial load and confounding factors (Table 3). We found significantly positive correlations between copy numbers of *P*. *gingivalis* and *Fretibacterium* sp. HOT 360 and percentage of ≥ 4 mm PPD (*b* = 0.15 and 0. 35, respectively); however, the copy number of *Fretibacterium* sp. HOT 360 showed a significantly positive correlation with percentage of BOP (b = 0.36). With respect to copy numbers of *TM7* sp. HOT 356, the confounding factors described in Table 3 did not show a significant correlation with periodontal parameters. Interestingly, only the increased amount of *Fretibacterium* sp. HOT 360 was significantly correlated with percentage ≥ 4 mm PPD and BOP.

**Table 3:** Correlation between periodontal parameters and bacterial load with adjusting confounders.

		Percentage of ≥ 4 mm PPD		Percentage of BOP	
Step		*b*	*P-value*	*b*	*P-value*
**1**	Constant				
	*P*. *gingivalis*	0.15 [0.14, 1.17]	0.02	0.09 [-0.28, 1.67]	NS
	*Fretibacterium* sp. HOT 360	0. 35 [0.67, 2.01]	< 0.01	0.36 [1.66, 4.13]	< 0.01
	*TM7 sp*. *HOT 356*	0.10 [-0.18, 1.12]	NS	0.09 [-0.53, 2.25]	NS
**2**	Constant				
	*P*. *gingivalis*	0.14 [0.05, 1.15]	0.04	0.11 [-0.22, 1.89]	NS
	*Fretibacterium* sp. HOT 360	0.32 [0.68, 2.02]	< 0.01	0.35 [1.46, 4.04]	< 0.01
	*TM7 sp*. *HOT 356*	0.13 [-0.09, 1.49]	NS	0.06 [-0.73, 1.94]	NS
	Gender	0.01 [−1.78, 2.03]	NS	0.02 [-0.14, 0.16]	NS
	Number of teeth	-0.04 [−0.26, 0.14]	NS	0.03 [-0.28, 0.49]	NS
	Age	-0.001 [-0.08, 0.09]	NS	0 [-0.34, 0.36]	NS
	Frequency of tooth brushing	-0.02 [-1.27, 0.97]	NS	-0.07 [-3.37, 0.89]	NS
	Duration of tooth brushing	0.55 [-0.26, 0.14]	NS	-0.01 [-0.18, 0.12]	NS
	Frequency of brush changing	0.03 [-0.87, 1.39]	NS	0.06 [-1.12, 3.10]	NS
	Use of special appliance	-0.07 [-0.91, 0.37]	NS	0.09 [-2.14, 6.10]	NS
	Smoking history	-0.07 [−2.36, 0.71]	NS	0.77 [-1.32, 4.89]	NS
	Alcohol consumption	-0.05 [-0.49, 0.22]	NS	-0.12 [-1.27, 0.01]	NS

Note: The data of multiple regression were demonstrated with beta [lower, upper of 95% confident interval value]

PPD: Step 1, R^2^ = 0.17; Step 2, ΔR^2^ = 0.14, BOP: Step 1, R^2^ = 0.18; Step 2, ΔR^2^ = 0.18

BOP, bleeding on brushing; PPD, probing pocket depth; NS, not significant.

### Difference in the bacterial loads of healthy and periodontitis groups with respect to each parameter

**Fig 2:**
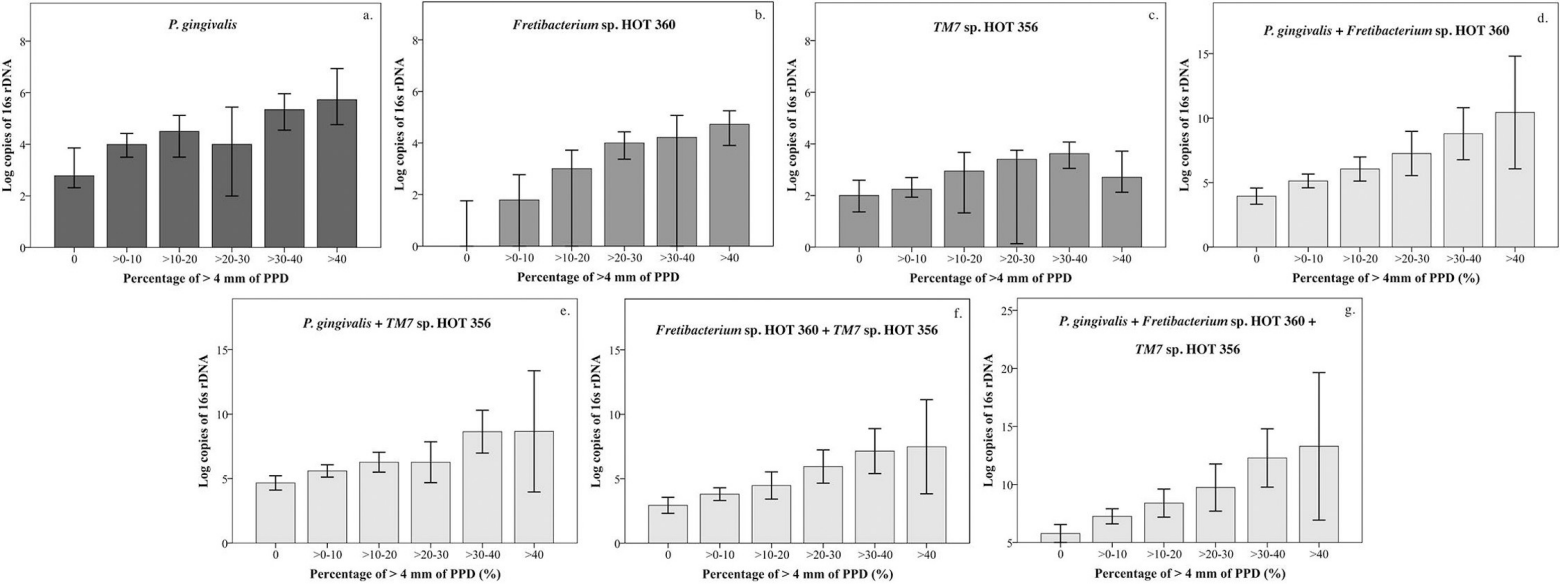
Bar graphs of bacterial loads and percentage of ≥4 mm groups. The abundance of ≥ 4 mm PPD, as log of rDNA (y-axis), were evaluated with respect to ranges showing 10 percent (x-axis). For non-parametric data, graphs are shown using mean and 95% confident interval of the mean (a to c). For non-parametric data, graphs are shown using median and 95% confident interval of the median (d to g). Significant differences were observed between the groups; data are demonstrated in S1 Table. The abundance of *Fretibacterium* sp. HOT 360 (b) and combination of bacterial groups with *Fretibacterium* sp. HOT 360 (d, f, and g) increased with increasing percentage of ≥ 4 mm PPD.

**Fig 3:**
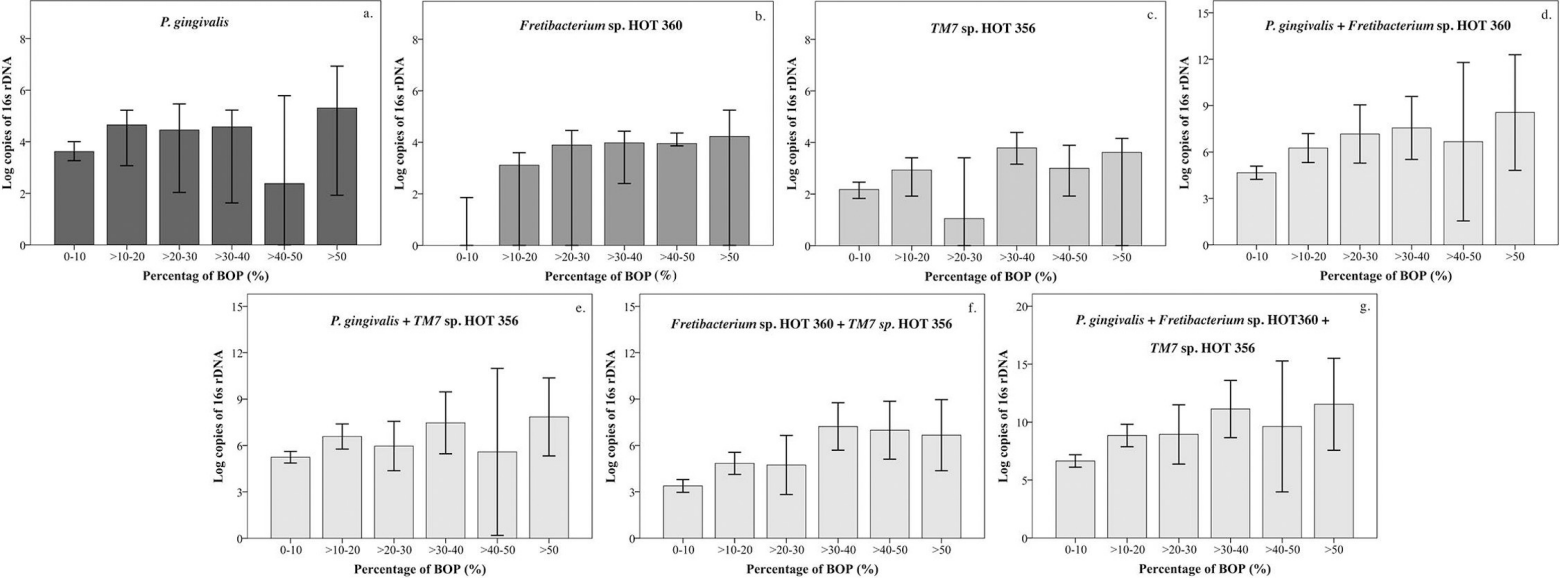
Bar graphs of bacterial loads and percentage of BOP. The bacteria were categorized according to Offenbacher’s classification. The percentage of BOP reflected the inflammatory status. The acceptable BOP, which could indicate an inflammatory lesion, was over 10%. Bar graphs show the abundance of bacterial species as log of rDNA (y-axis) with respect to 10 percent increases in BOP (x-axis). Parametric data are presented as mean and 95% confident interval of the mean (a to c). Non-parametric data are shown using median and 95% confident interval of the median (d to g). Results indicate significant differences between the groups. Data are demonstrated in S2 Table. The copy numbers of *Fretibacterium* sp. HOT 360 (b) were related to the percentage of BOP.

In the multiple regression analysis between periodontal parameters as a percentage of ≥4 mm and bacterial loads, only the bacterial load of *Fretibacterium* sp. *HOT 360* showed a significantly positive correlation with periodontal parameters. Therefore, we re-arranged the data and analyzed the difference between bacterial load and the range of percentage of periodontal parameters. The abundance of each bacterial species of as ≥4 mm PPD and BOP was categorized into 10 percent ranges, and the results are demonstrated in Figs 2 and 3 and Tables 4 and 5, respectively. Significant differences were observed for all bacterial species, including the percentages of ≥4 mm PPD and BOP (S1 and S2 Tables).

**Table 4:** The bacterial load of each species increased in groups of ≥4 mm PPD.

Bacterial species	Percent of >4 mm of PPD (%)						
	0 (n = 60)	> 0–10 (n = 97)	> 10–20 (n = 32)	> 20–30 (n = 15)	> 30–40 (n = 10)	> 40 (n = 3)	*P-value*
*P*. *gingivalis*	2.78 [2.32,2.86]	4.00 [3.50,4.42]	4.50 [3.505.12]	4.00 [2.00,5.44]	5.35 [4.55,5.96]	5.73 [4.76,6.94]	<0.01
*Fretibacterium* sp. HOT 360	0 [0,1.76]	1.80 [0,2.77]	3.01 [0, 3.73]	4.00 [3.37,4.43]	4.22 [0,5.07]	4.72 [3.91,5.25]	<0.01
*TM7* sp. HOT 356	2.0 [1.37,2.59]	2.25 [1.93,2.69]	2.95 [1.33,3.67]	3.40 [0.13,3.76]	3.62 [3.05,4.07]	2. 72 [2.13,3.72]	0.01
*P*. *gingivalis + Fretibacterium* sp. HOT 360	3.95 [3.32,4.57]	5.13 [4.59,5.66]	6.05 [5.12,6.99]	7.26 [5.54,8.97]	8.79 [6.77,10.81]	10.43 [6.07,14.80]	<0.01
*P*. *gingivalis + TM7* sp. HOT 356	4.67 [4.11,5.22]	5.59 [5.11,6.07]	6.27 [5.50,7.04]	6.27 [4.69,7.85]	8.64 [6.98,10.30]	8.66 [3.96,13.36]	<0.01
*TM7* sp. HOT 356 *+ Fretibacterium* sp. HOT 360	2.94 [2.32,3.56]	3.80 [3.31,4.29]	4.47 [3.41,5.53]	5.95 [4.66,7.24]	7.14 [5.40,8.88]	7.48 [3.83,11.13]	<0.01
*P*. *gingivalis + Fretibacterium* sp. HOT 360 + *TM7* sp. HOT 356	5.78 [5.00,6.55]	7.26 [6.61,7.90]	8.40 [7.19,9.61]	9.74 [7.70,11.77]	12.29 [9.77,14.80]	13.29 [6.94,9.64]	<0.01

**Table 5:** The bacterial loads of each species increased with increasing BOP.

Bacteria species	Percentage of BOP (%)						
	0–10 (n = 152)	> 10–20 (n = 32)	> 20–30 (n = 13)	> 30–40 (n = 10)	>40–50 (n = 4)	>50 (n = 6)	*P-value*
*P*. *gingivalis*	3.61 [3.27,4.01]	4.67 [3.01,5.22]	4.46 [2.04,5.47]	4.58 [1.63,5.23]	2.38 [0.00,5.79]	5.31 [1.93,6.94]	<0.05
*Fretibacterium* sp. HOT 360	0 [0.00,1.86]	3.12 [0.00, 3.60]	3.89 [0.00, 4.46]	3.98 [2.40,4.43]	3.95 [3.86,4.36]	4.23 [0.00,5.25]	<0.01
*TM7* sp. HOT 356	2.18 [1.83,2.46]	2.93 [1.92,3.40]	1.05 [0.00,3.40]	3.79 [3.16,4.39]	3.00 [1.92,3.90]	3.62 [0.00,4.16]	<0.01
*P*. *gingivalis + Fretibacterium* sp. HOT 360	4.66 [4.23,5.09]	6.26 [5.32,7.19]	7.17 [5.28,9.05]	7.56 [5.52,9.59]	6.67 [1.55,11.79]	8.56 [4.82,12.29]	<0.01
*P*. *gingivalis + TM7* sp. HOT 356	5.24 [4.86,5.62]	6.58 [5.77,7.39]	5.97 [4.37,7.56]	7.47 [5.47,9.46]	5.59 [0.19,10.99]	7.85 [5.33,10.37]	<0.01
*TM7* sp. HOT 356 *+ Fretibacterium* sp. HOT 360	3.38 [2.97,3.80]	4.84 [4.13,5.56]	4.74 [2.83,6.65]	7.23 [5.69,8.76]	6.67 [5.11,8.86]	6.67 [4.37,8.97]	<0.01
*P*. *gingivalis + Fretibacterium* sp. HOT 360 + *TM7* sp. HOT 356	6.65 [6.11,7.18]	8.84 [7.87,9.81]	8.94 [6.38,11.49]	11.12 [8.66,13.59]	9.62 [3.98,15.27]	11.54 [7.56,15.51]	<0.01

**Table 4** demonstrates the abundance of each bacterial species individually and in combination, which were plotted in Fig 2. All data were transformed to logarithmic values. Non-parametric data were analyzed by Kruskal–Wallis and Mann–Whitney *U* tests and are expressed as median [lower and upper bound of 95% confidence interval of the median]. Parametric data were analyzed by one-way ANOVA (Hochberg’s GT2) and are expressed as mean [lower and upper bound of 95% confident interval of the mean]. Significant differences were observed between groups and the results are presented in S1 Table.

**Table 5**demonstrates each bacterial species plotted in Fig 3 individually and in combination. Non-parametric data were analyzed by Kruskal–Wallis and Mann–Whitney *U* tests and are shown as median [lower and upper bound of 95% confident interval of the median]. Parametric data were analyzed by one-way ANOVA (Hochberg’s GT2) and are shown as mean [lower and upper bound of 95% confidence interval of the mean]. Significant differences were observed and the results are shown in S2 Table.

## Discussion

Recently, saliva screening is a convenient and non-invasive method that is becoming more common in oral disease studies, and in screening for dental caries and periodontitis. The subgingival plaque or gingival crevicular fluid were reported as the samples for evaluated the periodontitis status but these techniques were not convenient for periodontitis screening in large community scale [25]. Moreover, saliva can provide a cost-effective approach for the screening of large populations [26]. Whole saliva can be collected under no stimulation, or using methods such as mechanical stimulated or administration of mild acids [27]. In this study, we collected stimulated saliva because it is more rapid and simpler for participants who were hyposalivation or xerostomia. However, the technique used to collect saliva can affect the levels of certain biomarkers. Some biomarkers for periodontitis, such as tumor necrosis factor-alpha, C-telopeptide pyridinoline cross-links of type I collagen, and receptor activator of nuclear factor kappa-B [28], are sensitive to salivary collecting techniques [29].

Offenbacher’s classification is used in routine clinical diagnosis of periodontal disease. The periodontal parameters used for categorization were ≥4 mm PPD and percentage of BOP [22]. In this study, we aimed to measure the periodontopathogens that colonized the periodontal pocket and to evaluate the periodontal and inflammatory status of the participants. In patients with a recent diagnosis of periodontitis and loss of periodontium but without any sign of inflammation (pocket formation and BOP) on the day of sample collection were categorized as healthy. Although clinical attachment loss (CAL) is important for diagnosing and monitoring periodontal status during and after treatment, it does not explain the inflammatory status in terms of healing without any periodontium restoration. Therefore, the data PPD data were sufficient for screening for periodontitis. Although Offenbacher’s classification does not offer an accurate diagnosis of periodontal disease, it is useful for studying disease severity in a large population with respect to the expression of periodontitis biomarkers [22].

In our study, the participants completed a survey querying their oral hygiene behavior. The results of this study indicate that oral hygiene behavior was not associated with presence of periodontitis. The etiology of periodontitis is associated with numerous factors, and oral hygiene behavior is only one of the risk factors for periodontitis [30].

Smoking is a cofactor that affects periodontal tissues and the inflammation process. Smoking can suppress bleeding from the periodontal pockets; this suppression continues after cessation of smoking. This is because agents in cigarettes influence vascular and cellular properties during the inflammatory process [31]. For this reason, using red blood cells or other inflammatory biomarkers in the blood vessels is inaccurate for evaluating the inflammatory status and development of periodontitis in smokers. Bacterial populations are not affected by smoking; this notion is supported by a report indicating that populations of periodontopathogens were not significantly different between smoking and nonsmoking patients with periodontitis [32]. Our results indicate that there were no significant differences in the bacterial loads of smokers and non-smokers with same periodontal conditions (S1 Fig). Moreover, in the additional data (S2 Fig), the periodontal conditions of individuals with same smoking behavior differed significantly, with the abundance of *P*. *gingivalis* (a-3) and *Fretibacterium* sp. HOT 360 (b-3) being lower in non-smoking healthy individuals than in non-smoking individuals with periodontitis. No differences were observed in bacterial load of TM7 sp. HOT 356 and smoking conditions with either periodontal conditions. Moreover, bacterial loads of *P*. *gingivalis* and Fretibacterium sp. HOT 360 which were categorized into current and stopped smoking with different periodontal conditions were also not significantly different. This result supports previous studies indicating that bacterial markers may be useful for periodontitis screening because they are not influenced by smoking [32]. However, several studies reported that smoking behavior was influent in periodontium tissue [33, 34] and oral microbiome [35, 36]. Further studies are needed to determine whether bacteriologic indicators can be used effectively as well as in smokers and in non-smokers.

*P*. *gingivalis* is a representative bacterial species in chronic periodontitis, and this species was strongly correlated with development of periodontitis [11]. Numerous studies show that virulent factors present in *P*. *gingivalis*, such as capsule, fimbriae, lipopolysaccharides, and collagenase, can stimulate the host immune response [37]. In 2012, Hajishengallis et al., reported that *P*. *gingivalis* is key in the pathogenesis of periodontitis as described by symbiosis-dysbiosis hypothesis [38]. For this reason, we used the growth of *P*. *gingivalis* as our control for comparison with the growth of *Fretibacterium* sp. HOT 360 and *TM7* sp. HOT 356. Several studies, however, have reported that *P*. *gingivalis* is commonly found in both healthy Asian adults and those with periodontitis [39]. Moreover, in 1998, Griffen et al. used q-PCR to show that *P*. *gingivalis* is present in 22% of healthy Caucasian and 38% of healthy African-American adults [40]. In 2013, Kato et al. also used PCR to show that *P*. *gingivalis* is present in 40% of healthy Japanese adults [41]. In our present study, the levels of *P*. *gingivalis* were significantly higher in participants with periodontitis (*P*< 0.05), and presence of *P*. *gingivalis* was significantly positively correlated with periodontal parameters as the percentage of ≥ 4 mm PPD and BOP (*P*< 0.05); however, *P*. *gingivalis* was also found in healthy participants.

The two uncultivable periodontal bacteria, *Fretibacterium* sp. HOT 360 and *TM7* sp. HOT 356, were selected from more than 400 bacterial species. These species were selected because they are found at high levels in deep periodontal pockets [14]. In our present study, the levels of *Fretibacterium* sp. HOT 360 were significantly higher in participants with periodontitis than in healthy controls. In participants with periodontitis, we found significant positive correlations between the levels of *Fretibacterium* sp. HOT 360 and percentage of ≥ 4 mm PPD and BOP. These findings agree with those of Oliveira et al., [16], who reported in 2016 that mean levels of *Fretibacterium* sp. HOT 360 were significantly higher in patients with periodontitis than healthy condition. Additionally, RNA-oligonucleotide quantification showed that higher levels of *Fretibacterium* sp. HOT 360 are significantly correlated with greater pocket depth [16].

Because *Fretibacterium* sp. HOT 360 could not be cultured, its characteristics and virulent factors are still unknown. However, our results are consistent with those reported by Brinig et al. [19], who showed that higher bacterial load of *TM7* sp. HOT 356 was observed in the subgingival plaque of patients in early stages of chronic periodontitis than in healthy controls.

The results of multiple regression analysis, and differences in bacterial loads within periodontal parameter groups are shown in Tables 3, 4 and 5 and Figs 2 and 3 and S1 and S2. The growth levels of each bacterial species were combined to show how a combined bacterial load can be used as a bacterial biomarker. However, our results indicated that with respect to each 10 percent increase in ≥ 4 mm PPD, only the bacterial loads of *Fretibacterial* sp. HOT 360 increased when combined with other bacterial species. Furthermore, multiple regression analysis demonstrated that only *Fretibacterium* sp. HOT 360 showed a significant correlation with percentage of BOP. However, some of the differences within the range of BOP subgroups were not significantly different (S2).

The periodontal parameters in Figs 2 and 3 and Tables 4 and 5 are arranged in 10 percent ranges because firstly, in the classification suggested by Offenbacher, more than 10% BOP was considered to indicate an inflammatory response. Secondly, *P*. *gingivalis*, a globally distributed periodontopathogen, did not show a positive correlation in the multiple regression analysis. Furthermore, this analysis revealed that the periodontal parameters in some 10 percent ranges were not significantly different because the sample size in each group was very small and unequal. In contrast, this demonstration could be easily understood which was similar as Percent of PPD. Additionally, we could not control the sample size in each group because of the limitation of time and number of participants. Therefore, to increase the reliability of these findings, studies using larger sample size and a more diverse group of participants in terms of disease severity are needed. Moreover, the statistical findings of this study must be interpreted with caution. Therefore, the further studies should be more controlled and better planned for protocol. However, although the results lack adequate reliability, this is the first report showing the presence of uncultivable periodontopathogens in salivary samples of periodontitis patients.

## Conclusions

In our study, some of patients presented with various depths of PPD but absence of BOP. Therefore, the data we were able to obtain with respect to percentage of BOP were scant and presented a limitation in this study. In our future studies on gingivitis, we intend to confirm that *Fretibacterium* sp. HOT 360, *P*. *gingivalis*, and *TM7* sp. HOT 356 are associated with BOP.

All the bacterial species examined in our study showed higher levels in the saliva of participants who were diagnosed with periodontitis. Moreover, *P*. *gingivalis* and *TM7* sp. HOT 356 were detected in most of the healthy participants in Figs 1, 2 and 3 (a. and c.). Conversely, *Fretibacterium* sp. HOT 360 was rarely detected in healthy participants. Moreover, *Fretibacterium* sp. HOT 360 was the only bacterial species that showed significantly increased levels in the periodontitis group, and significantly positively correlated with ≥ 4 mm PPD and BOP. Further studies of bacterial biomarkers in saliva will help refine this method for periodontitis screening. Because this approach is not invasive and straightforward, it can be widely used for screening individuals as well as large communities. Periodontopathogens are still important bacterial biomarkers because they have been extensively studied. However, some of the cultivable periodontopathogens are found in healthy adult, while uncultivable bacterial species, such as *Fretibacterium* sp. HOT 360, can be more selectively used for periodontitis screening. The bacterial species examined in this study were detected at significantly higher levels in participants with periodontitis compared with the levels observed in healthy participants. Moreover, multiple regression analysis indicated that *Fretibacterium* sp. HOT 360 showed a positive correlation with the clinical periodontal parameters ≥ 4 mm PPD and BOP. Our results indicate that uncultivable bacteria *Fretibacterium* sp. HOT 360, which is present in saliva, can be used as one of bacterial biomarker for periodontitis screening.

## Supporting information

S1 Table Comparison of bacterial loads between groups of ≥4 mm PPD (%). - ✓ Significant difference.
- X No significant difference.
(DOCX)

S2 Table Comparison of bacterial load between groups with BOP (%). - ✓ Significant difference.
- X No significant difference.
(DOCX)

S1 Fig Bacterial loads with respect to smoking status in different participants with varying severity of periodontitis. Box plot graphs demonstrated bacterial loads of the different smoking status with same periodontal conditions. The amount of bacterial species as *P*. *gingivalis* (a and d), *Fretibacterium* sp. HOT 360 (b and e) and *TM7* sp. HOT 356 (c and f) were demonstrated in Y-axis and smoking status with different periodontal conditions were demonstrated in X-axis. The data were analyzed by Kolmogorov–Smirnov test, Kruskal–Wallis and Mann–Whitney *U* tests, respectively. The results revealed that the significant difference was noticed between bacterial loads of *P*. *gingivalis* (a and d), *Fretibacterium* sp. HOT 360 (b and e) and *TM7* sp. HOT 356. (c. and f.), P = 0.05.
(TIF)

S2 Fig Bacterial loads with respect to periodontal conditions in different smoking status. Box plot graphs demonstrated bacterial loads of the same smoking status with different periodontal conditions. The amount of bacterial species as *P*. *gingivalis* (a-1 to a-3), *Fretibacterium* sp. HOT 360 (b-1 to b-3) and *TM7* sp. HOT 356 (c-1 to c-3) were demonstrated in Y-axis and smoking status with different periodontal conditions were demonstrated in X-axis. The data were analyzed by Kolmogorov–Smirnov test, Kruskal–Wallis and Mann–Whitney *U* tests, respectively. The results revealed that the significant difference was noticed between bacterial loads of *P*. *gingivalis* (a-3.) and *Fretibacterium* sp. HOT 360 (c-3) which were healthy condition with non-smoking and non-smoking with periodontitis condition, *P = 0.05. Refer to S1 and S2 Figs ƒ of this study, smoking behavior was not influents the periodontitis screening.
(TIF)

## References

[1] Offenbacher S. Periodontal diseases: pathogenesis. Ann Periodontol. 1996;1(1):821–78. 10.1902/annals.1996.1.1.821 . 9118282

[2] Kim JJ, Kim CJ, Camargo PM. Salivary biomarkers in the diagnosis of periodontal diseases. J Calif Dent Assoc. 2013;41(2):119–24. Epub 2013/03/20. 23505757 PMC3629836

[3] Giannobile WV, Beikler T, Kinney JS, Ramseier CA, Morelli T, Wong DT. Saliva as a diagnostic tool for periodontal disease: current state and future directions. Periodontology 2000. 2009;50(1):52–64. 10.1111/j.1600-0757.2008.00288.x 19388953 PMC5695225

[4] Zhang L, Henson BS, Camargo PM, Wong DT. The clinical value of salivary biomarkers for periodontal disease. Periodontol 2000. 2009;51:25–37. 10.1111/j.1600-0757.2009.00315.x . 19878467

[5] Paju S, Pussinen PJ, Suominen-Taipale L, Hyvönen M, Knuuttila M, Könönen E. Detection of Multiple Pathogenic Species in Saliva Is Associated with Periodontal Infection in Adults. Journal of Clinical Microbiology. 2009;47(1):235–8. 10.1128/JCM.01824-08 PubMed PMID: PMC2620834. 19020069 PMC2620834

[6] Page RC, Schroeder HE. Pathogenesis of inflammatory periodontal disease. A summary of current work. Lab Invest. 1976;34(3):235–49. . 765622

[7] Socransky SS, Haffajee AD, Cugini MA, Smith C, Kent RL Jr. Microbial complexes in subgingival plaque. J Clin Periodontol. 1998;25(2):134–44. . 9495612 10.1111/j.1600-051x.1998.tb02419.x

[8] Holt SC, Ebersole JL. Porphyromonas gingivalis, Treponema denticola, and Tannerella forsythia: the "red complex", a prototype polybacterial pathogenic consortium in periodontitis. Periodontol 2000. 2005;38:72–122. 10.1111/j.1600-0757.2005.00113.x . 15853938

[9] Dewhirst FE, Chen T, Izard J, Paster BJ, Tanner AC, Yu WH, et al The human oral microbiome. J Bacteriol. 2010;192(19):5002–17. 10.1128/JB.00542-10 20656903 PMC2944498

[10] Paster BJ, Olsen I, Aas JA, Dewhirst FE. The breadth of bacterial diversity in the human periodontal pocket and other oral sites. Periodontol 2000. 2006;42:80–7. 10.1111/j.1600-0757.2006.00174.x . 16930307

[11] Salminen A, Kopra KA, Hyvarinen K, Paju S, Mantyla P, Buhlin K, et al Quantitative PCR analysis of salivary pathogen burden in periodontitis. Front Cell Infect Microbiol. 2015;5:69 10.3389/fcimb.2015.00069 26484315 PMC4589666

[12] Maruyama N, Maruyama F, Takeuchi Y, Aikawa C, Izumi Y, Nakagawa I. Intraindividual variation in core microbiota in peri-implantitis and periodontitis. Sci Rep. 2014;4:6602 10.1038/srep06602 25308100 PMC4194447

[13] Abusleme L, Dupuy AK, Dutzan N, Silva N, Burleson JA, Strausbaugh LD, et al The subgingival microbiome in health and periodontitis and its relationship with community biomass and inflammation. ISME J. 2013;7(5):1016–25. 10.1038/ismej.2012.174 23303375 PMC3635234

[14] Perez-Chaparro PJ, Goncalves C, Figueiredo LC, Faveri M, Lobao E, Tamashiro N, et al Newly identified pathogens associated with periodontitis: a systematic review. J Dent Res. 2014;93(9):846–58. 10.1177/0022034514542468 25074492 PMC4541103

[15] You M, Mo S, Watt RM, Leung WK. Prevalence and diversity of Synergistetes taxa in periodontal health and disease. J Periodontal Res. 2013;48(2):159–68. 10.1111/j.1600-0765.2012.01516.x . 22881378

[16] Oliveira RR, Fermiano D, Feres M, Figueiredo LC, Teles FR, Soares GM, et al Levels of Candidate Periodontal Pathogens in Subgingival Biofilm. J Dent Res. 2016;95(6):711–8. 10.1177/0022034516634619 26936213 PMC4924544

[17] Kirst ME, Li EC, Alfant B, Chi YY, Walker C, Magnusson I, et al Dysbiosis and alterations in predicted functions of the subgingival microbiome in chronic periodontitis. Appl Environ Microbiol. 2015;81(2):783–93. 10.1128/AEM.02712-14 25398868 PMC4277562

[18] Soro V, Dutton LC, Sprague SV, Nobbs AH, Ireland AJ, Sandy JR, et al Axenic culture of a candidate division TM7 bacterium from the human oral cavity and biofilm interactions with other oral bacteria. Appl Environ Microbiol. 2014;80(20):6480–9. 10.1128/AEM.01827-14 25107981 PMC4178647

[19] Brinig MM, Lepp PW, Ouverney CC, Armitage GC, Relman DA. Prevalence of bacteria of division TM7 in human subgingival plaque and their association with disease. Appl Environ Microbiol. 2003;69(3):1687–94. 10.1128/AEM.69.3.1687-1694.2003 12620860 PMC150096

[20] Kumar PS, Griffen AL, Barton JA, Paster BJ, Moeschberger ML, Leys EJ. New bacterial species associated with chronic periodontitis. J Dent Res. 2003;82(5):338–44. 10.1177/154405910308200503 . 12709498

[21] Sunaga M, Kondo K, Adachi T, Miura Y, Kinoshita A. Development and evaluation of a new dental model at Tokyo Medical and Dental University for the practice of periodontal pocket probing. J Dent Educ. 2013;77(9):1185–92. Epub 2013/09/05. . 24002857

[22] Offenbacher S, Barros SP, Singer RE, Moss K, Williams RC, Beck JD. Periodontal disease at the biofilm-gingival interface. J Periodontol. 2007;78(10):1911–25. Epub 2007/12/07. 10.1902/jop.2007.060465 . 18062113

[23] Polgarova K, Behuliak M, Celec P. Effect of saliva processing on bacterial DNA extraction. New Microbiol. 2010;33(4):373–9. . 21213596

[24] Amodini Rajakaruna G, Umeda M, Uchida K, Furukawa A, Yuan B, Suzuki Y, et al Possible translocation of periodontal pathogens into the lymph nodes draining the oral cavity. J Microbiol. 2012;50(5):827–36. 10.1007/s12275-012-2030-8 . 23124752

[25] Javaid MA, Ahmed AS, Durand R, Tran SD. Saliva as a diagnostic tool for oral and systemic diseases. J Oral Biol Craniofac Res. 2016;6(1):66–75. Epub 2016/03/05. 10.1016/j.jobcr.2015.08.006 26937373 PMC4756071

[26] Kononen E, Paju S, Pussinen PJ, Hyvonen M, Di Tella P, Suominen-Taipale L, et al Population-based study of salivary carriage of periodontal pathogens in adults. J Clin Microbiol. 2007;45(8):2446–51. 10.1128/JCM.02560-06 17567788 PMC1951210

[27] Belstrom D, Holmstrup P, Bardow A, Kokaras A, Fiehn NE, Paster BJ. Comparative analysis of bacterial profiles in unstimulated and stimulated saliva samples. J Oral Microbiol. 2016;8:30112 Epub 2016/03/19. 10.3402/jom.v8.30112 26987356 PMC4796727

[28] Stathopoulou PG, Buduneli N, Kinane DF. Systemic Biomarkers for Periodontitis. Current Oral Health Reports. 2015;2(4):218–26. 10.1007/s40496-015-0072-9

[29] Schafer CA, Schafer JJ, Yakob M, Lima P, Camargo P, Wong DT. Saliva diagnostics: utilizing oral fluids to determine health status. Monogr Oral Sci. 2014;24:88–98. Epub 2014/05/28. 10.1159/000358791 . 24862597

[30] Genco RJ, Borgnakke WS. Risk factors for periodontal disease. Periodontol 2000. 2013;62(1):59–94. Epub 2013/04/12. 10.1111/j.1600-0757.2012.00457.x . 23574464

[31] Sreedevi M, Ramesh A, Dwarakanath C. Periodontal status in smokers and nonsmokers: a clinical, microbiological, and histopathological study. Int J Dent. 2012;2012:571590 10.1155/2012/571590 22505904 PMC3296295

[32] Mager DL, Haffajee AD, Socransky SS. Effects of periodontitis and smoking on the microbiota of oral mucous membranes and saliva in systemically healthy subjects. Journal of Clinical Periodontology. 2003;30(12):1031–7. 10.1046/j.0303-6979.2003.00418.x 15002888

[33] Mullally BH. The influence of tobacco smoking on the onset of periodontitis in young persons. Tob Induc Dis. 2004;2(2):53–65. Epub 2004/01/01. 10.1186/1617-9625-2-2-53 19570272 PMC2671536

[34] Cesar Neto JB, Rosa EF, Pannuti CM, Romito GA. Smoking and periodontal tissues: a review. Braz Oral Res. 2012;26 Suppl 1:25–31. Epub 2013/01/18. . 23318741 10.1590/s1806-83242012000700005

[35] Kumar PS, Matthews CR, Joshi V, de Jager M, Aspiras M. Tobacco smoking affects bacterial acquisition and colonization in oral biofilms. Infect Immun. 2011;79(11):4730–8. Epub 2011/08/24. 10.1128/IAI.05371-11 21859855 PMC3257914

[36] Kubota M, Tanno-Nakanishi M, Yamada S, Okuda K, Ishihara K. Effect of smoking on subgingival microflora of patients with periodontitis in Japan. BMC Oral Health. 2011;11:1 Epub 2011/01/07. 10.1186/1472-6831-11-1 21208407 PMC3020163

[37] How KY, Song KP, Chan KG. Porphyromonas gingivalis: An Overview of Periodontopathic Pathogen below the Gum Line. Front Microbiol. 2016;7:53 Epub 2016/02/24. 10.3389/fmicb.2016.00053 26903954 PMC4746253

[38] Hajishengallis G, Darveau RP, Curtis MA. The keystone-pathogen hypothesis. Nat Rev Microbiol. 2012;10(10):717–25. 10.1038/nrmicro2873 22941505 PMC3498498

[39] Torrungruang K, Jitpakdeebordin S, Charatkulangkun O, Gleebbua Y. Porphyromonas gingivalis, Aggregatibacter actinomycetemcomitans, and Treponema denticola / Prevotella intermedia Co-Infection Are Associated with Severe Periodontitis in a Thai Population. PLoS One. 2015;10(8):e0136646 Epub 2015/08/28. 10.1371/journal.pone.0136646 26313005 PMC4552424

[40] Griffen AL, Becker Mr Fau—Lyons SR, Lyons Sr Fau—Moeschberger ML, Moeschberger Ml Fau—Leys EJ, Leys EJ. Prevalence of Porphyromonas gingivalis and periodontal health status. (0095–1137 (Print)). 10.1128/jcm.36.11.3239-3242.1998 PMC105308 9774572

[41] Kato A, Imai K Fau—Ochiai K, Ochiai K Fau—Ogata Y, Ogata Y. Higher prevalence of Epstein-Barr virus DNA in deeper periodontal pockets of chronic periodontitis in Japanese patients. (1932–6203 (Electronic)). 10.1371/journal.pone.0071990 PMC3753341 23991022

