# Supplementary material for: Fretibacterium sp. human oral taxon 360 is a novel biomarker for periodontitis screening in the Japanese population
Source: PLoS One. 2019 Jun 19;14(6):e0218266. doi: 10.1371/journal.pone.0218266 (PMC6584019; doi:10.1371/journal.pone.0218266)
Supplement: S1 Table — - ✓ Significant difference. - X No significant difference. (DOCX) [file pone.0218266.s001.docx]

**S1 table** : The comparison of bacterial load between groups of ≥4 mm of PPD (%)

| Bacterial species  Comparison  between groups  of ≥ 4 mm PPD (%) | *P. gingivalis* | *Fretibacterium* sp. HOT 360 | *TM7* sp. HOT 356 | *P. gingivalis* + *Fretibacterium* sp. HOT 360 | *P. gingivalis* + *TM7* sp. HOT 356 | *TM7* sp. HOT 356 + *Fretibacterium* sp. HOT 360 | *P. gingivalis* + *Fretibacterium* sp. HOT 360+ *TM7* sp. HOT 356 |
| --- | --- | --- | --- | --- | --- | --- | --- |
| 0/ > 0-10 | ✓ | ✓ | X | X | X | X | X |
| 0/ >10-20 | ✓ | ✓ | X | ✓ | ✓ | X | ✓ |
| 0/ >20-30 | X | ✓ | X | ✓ | X | ✓ | ✓ |
| 0/ >30-40 | ✓ | ✓ | ✓ | ✓ | ✓ | ✓ | ✓ |
| 0/ >40 | ✓ | ✓ | X | ✓ | X | ✓ | ✓ |
| > 0-10/ >10-20 | X | X | X | X | X | X | X |
| > 0-10/ >20-30 | X | X | X | X | X | ✓ | ✓ |
| > 0-10/ >30-40 | ✓ | ✓ | ✓ | ✓ | ✓ | ✓ | ✓ |
| > 0-10/ >40 | ✓ | ✓ | X | ✓ | X | X | X |
| >10-20/ >20-30 | X | ✓ | X | X | X | X | X |
| >10-20/ >30-40 | ✓ | ✓ | X | X | X | ✓ | ✓ |
| >10-20/ >40 | ✓ | ✓ | X | X | X | X | X |
| >20-30/ >30-40 | X | X | X | X | X | X | X |
| >20-30/ >40 | X | X | X | X | X | X | X |
| >30-40/ >40 | X | X | X | X | X | X | X |

- ✓ Significant difference

- X Non-significant difference
