# Supplementary material for: Fretibacterium sp. human oral taxon 360 is a novel biomarker for periodontitis screening in the Japanese population
Source: PLoS One. 2019 Jun 19;14(6):e0218266. doi: 10.1371/journal.pone.0218266 (PMC6584019; doi:10.1371/journal.pone.0218266)
Supplement: S2 Table — - ✓ Significant difference. - X No significant difference. (DOCX) [file pone.0218266.s002.docx]

**S2 Table:** The comparison of bacterial load between groups of BOP (%)

| Bacterial species  Comparison between groups  of BOP (%) | *P. gingivalis* | *Fretibacterium* sp. HOT 360 | *TM7* sp. HOT 356 | *P. gingivalis* + *Fretibacterium* sp. HOT 360 | *P. gingivalis* + *TM7* sp. HOT 356 | *TM7* sp. HOT 356 + *Fretibacterium* sp. HOT 360 | *P. gingivalis* + *Fretibacterium* sp. HOT 360+ *TM7* sp. HOT 356 |
| --- | --- | --- | --- | --- | --- | --- | --- |
| 0-10/ >10-20 | ✓ | ✓ | ✓ | ✓ | X | ✓ | ✓ |
| 0-10/ >20-30 | X | ✓ | X | ✓ | X | X | X |
| 0-10/ >30-40 | X | ✓ | ✓ | ✓ | X | ✓ | ✓ |
| 0-10/ >40-50 | X | ✓ | X | X | X | X | X |
| 0-10/ >50 | ✓ | ✓ | X | ✓ | X | ✓ | ✓ |
| >10-20/ >20-30 | X | X | X | X | X | X | X |
| >10-20/ >30-40 | X | ✓ | ✓ | X | X | X | X |
| >10-20/ >40-50 | X | ✓ | X | X | X | X | X |
| >10-20/ >50 | X | ✓ | X | X | X | X | X |
| >20-30/ >30-40 | X | X | ✓ | X | X | X | X |
| >20-30/ >40-50 | X | X | X | X | X | X | X |
| >20-30/ >50 | X | X | X | X | X | X | X |
| >30-40/ >40-50 | X | X | X | X | X | X | X |
| >30-40/ >50 | X | X | X | X | X | X | X |
| >40-50/ >50 | X | X | X | X | X | X | X |

- ✓ Significant difference

- X Non-significant difference
