# Supplementary figures and images for: Fretibacterium sp. human oral taxon 360 is a novel biomarker for periodontitis screening in the Japanese population
Source: PLoS One. 2019 Jun 19;14(6):e0218266. doi: 10.1371/journal.pone.0218266 (PMC6584019; doi:10.1371/journal.pone.0218266)

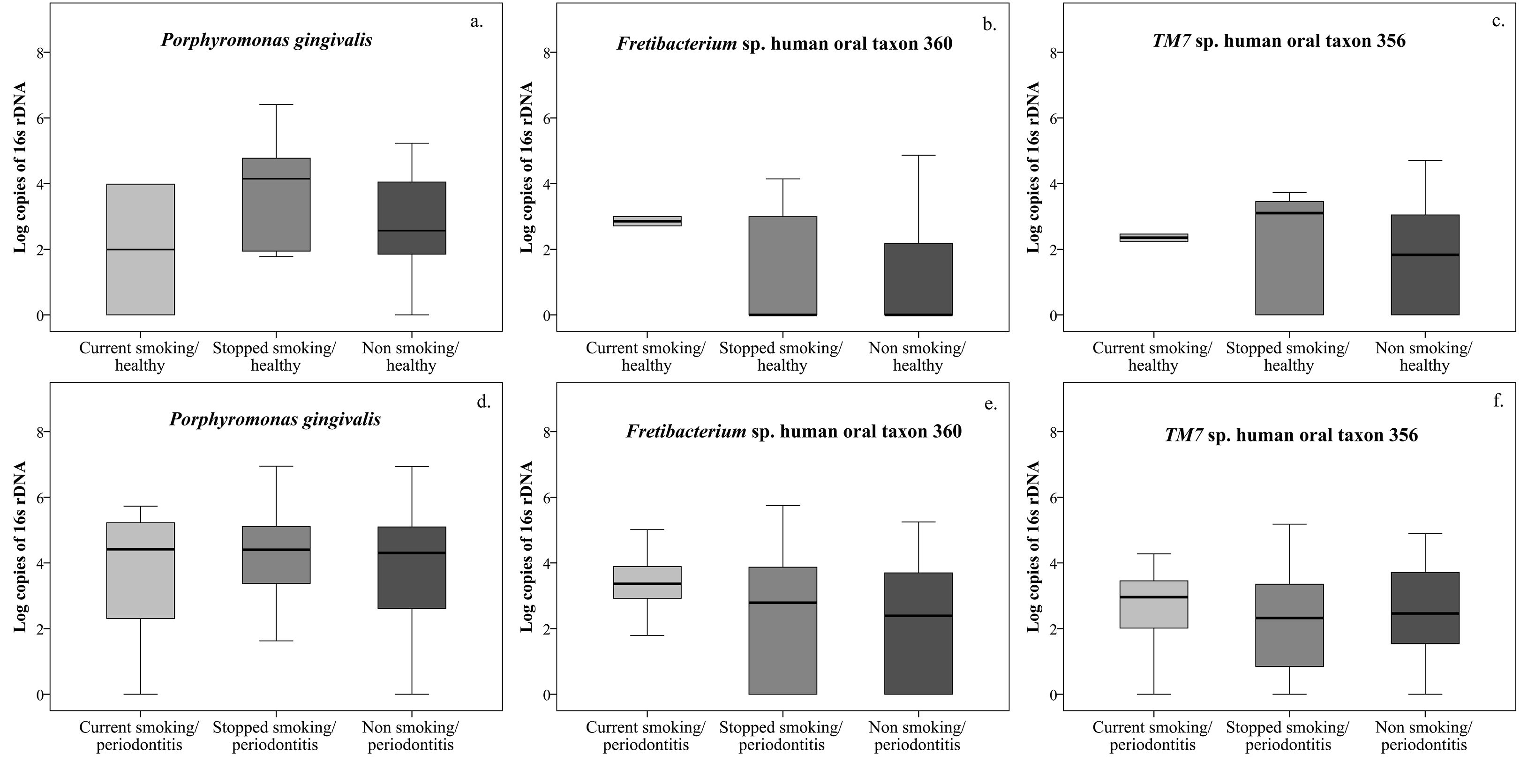

Supplement: S1 Fig — Box plot graphs demonstrated bacterial loads of the different smoking status with same periodontal conditions. The amount of bacterial species as P. gingivalis (a and d), Fretibacterium sp. HOT 360 (b and e) and TM7 sp. HOT 356 (c and f) were demonstrated in Y-axis and smoking status with different periodontal conditions were demonstrated in X-axis. The data were analyzed by Kolmogorov–Smirnov test, Kruskal–Wallis and Mann–Whitney U tests, respectively. The results revealed that the significant difference was noticed between bacterial loads of P. gingivalis (a and d), Fretibacterium sp. HOT 360 (b and e) and TM7 sp. HOT 356. (c. and f.), P = 0.05. (TIF) [file pone.0218266.s003.tif]

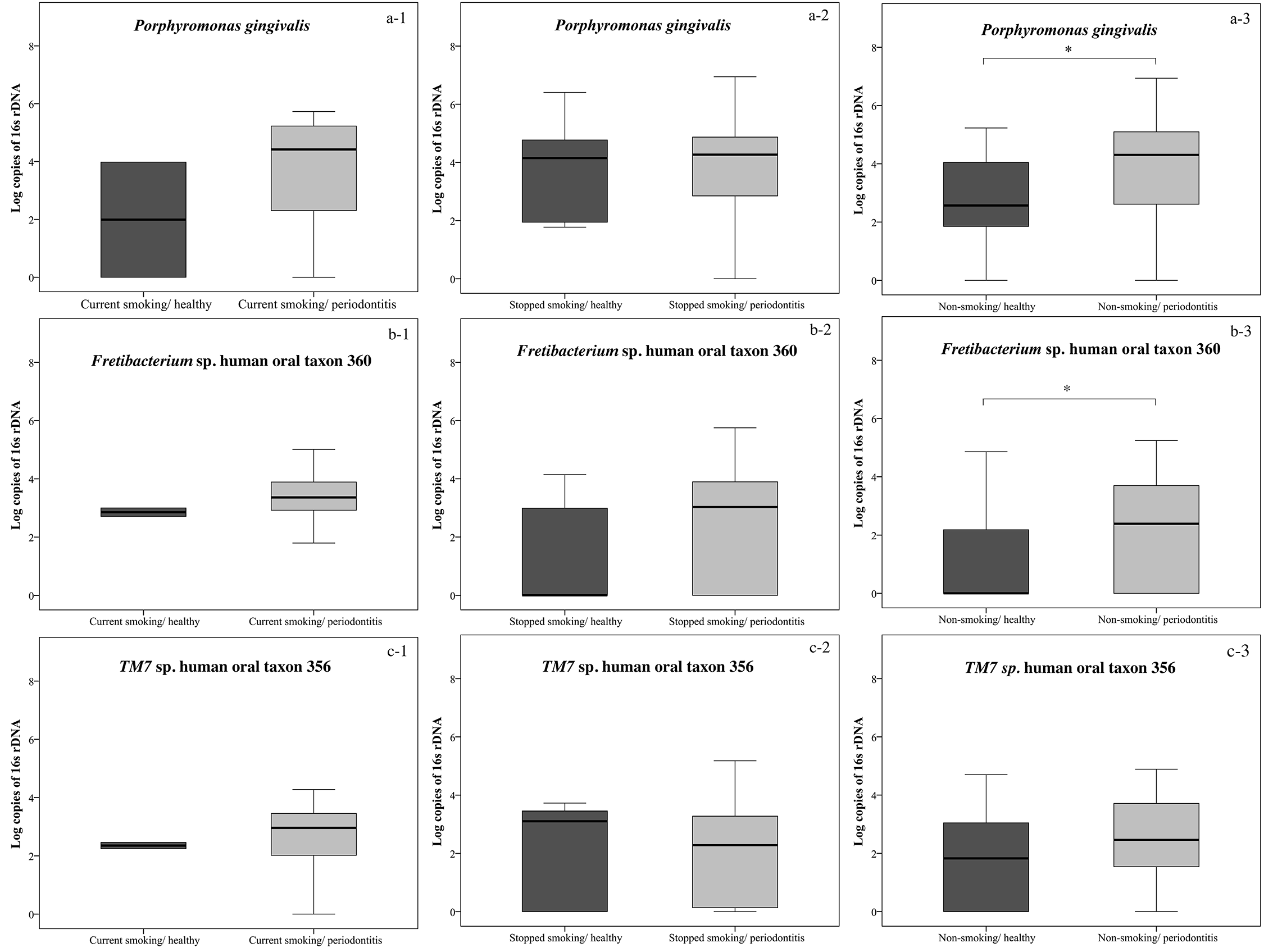

Supplement: S2 Fig — Box plot graphs demonstrated bacterial loads of the same smoking status with different periodontal conditions. The amount of bacterial species as P. gingivalis (a-1 to a-3), Fretibacterium sp. HOT 360 (b-1 to b-3) and TM7 sp. HOT 356 (c-1 to c-3) were demonstrated in Y-axis and smoking status with different periodontal conditions were demonstrated in X-axis. The data were analyzed by Kolmogorov–Smirnov test, Kruskal–Wallis and Mann–Whitney U tests, respectively. The results revealed that the significant difference was noticed between bacterial loads of P. gingivalis (a-3.) and Fretibacterium sp. HOT 360 (c-3) which were healthy condition with non-smoking and non-smoking with periodontitis condition, *P = 0.05. Refer to S1 and S2 Figs ƒ of this study, smoking behavior was not influents the periodontitis screening. (TIF) [file pone.0218266.s004.tif]
